# Glucosyltransferase activity-based screening identifies tannic acid as an inhibitor of *Streptococcus mutans* biofilm

**DOI:** 10.3389/fmicb.2025.1555497

**Published:** 2025-03-27

**Authors:** Mai Xu, Xinyan Wang, Tao Gong, Ziyi Yang, Dian Zhang, Qizhao Ma, Yuqing Li

**Affiliations:** ^1^State Key Laboratory of Oral Diseases, National Clinical Research Center for Oral Diseases, West China Hospital of Stomatology, Sichuan University, Chengdu, China; ^2^Center for Archaeological Science, Sichuan University, Chengdu, China

**Keywords:** dental caries, *Streptococcus mutans*, glucosyltransferase, glucan, tannic acid

## Abstract

Dental caries is a biofilm-related chronic infectious disease. *Streptococcus mutans* is the core microorganism that leads to caries, with its capacity to form biofilms via glucosyltransferases (Gtfs) being the predominant virulence factor contributing to this condition. Therefore, researching novel drugs targeting Gtf is important for treating dental caries. Our study established a rapid detection method for Gtf activity to screen over 1,000 compounds from the Selleck Natural Product Library. We identified tannic acid (TA) as a potential inhibitor of Gtf activity. *In vitro* experiments suggested that TA could inhibit extracellular polysaccharide (EPS) synthesis and biofilm formation in *S. mutans* by selectively antagonizing Gtf rather than directly killing the bacteria. Molecular docking experiments confirmed a strong binding affinity between TA and Gtf. In summary, TA exhibits good anti-virulence performance against *S. mutans*, indicating its potential value in anti-biofilm and anti-caries applications.

## 1 Introduction

Dental caries, a chronic infectious disease affecting the hard tissues of the teeth, is influenced by several factors, primarily bacteria. It is one of the most prevalent health conditions affecting people of all ages (Petersen and Kwan, 2009). Caries can cause pain and discomfort, and when the infection spreads to the pulp, it can lead to severe pain and periapical infections, resulting in tooth loss and even systemic diseases, thereby imposing a huge burden on individuals and society (Pitts et al., 2017).

Dental plaque biofilm is the initiating factor in the development of caries. It is a structured microbial community attached to the tooth surface and encased in an extracellular matrix (Giacaman, 2018). The oral microbiota often maintains a dynamic balance with the host. During disruptions caused by host or environmental factors, shifting the microbial community from a physiological to a pathological composition, such as increased acid-producing and acid-tolerant microbes, can promote demineralization of the tooth structure, eventually causing caries (Marsh and Zaura, 2017). *S. mutans*, a member of the endogenous oral flora, plays a key role in the pathogenesis of dental caries (Bowden and Li, 1997; Ben-Zaken et al., 2021). It can produce and tolerate acids, utilizing sucrose to synthesize extracellular polysaccharides (EPS) through glucosyltransferases (Gtfs). Additionally, it can adhere to glucan-coated tooth surfaces, enabling it to effectively colonize the oral cavity and form a highly cariogenic dental biofilm (Marsh and Bradshaw, 1995; Loesche, 1986; Hasan et al., 2012).

*S. mutans* produces three types of Gtf enzymes: GtfB, GtfC, and GtfD (Koo et al., 2002). GtfB and GtfC rapidly utilize sucrose to synthesize insoluble and soluble glucans on the bacterial surface, including bacteria that do not produce Gtf. GtfD-produced soluble glucans serve as primers for GtfB, enhancing the overall synthesis of EPS (Bowen and Koo, 2011). Additionally, EPS, serving as an adhesive for bacterial attachment and a three-dimensional framework for dental biofilms, provides a unique microenvironment for microbial growth, metabolism, and survival. This allows microbes to develop stronger resistance to harsh and challenging environmental conditions, host immunity, and traditional antimicrobial therapies, thus preventing disintegration and enhancing their mechanical stability (Mattos-Graner et al., 2004; Bowen et al., 2018). Therefore, targeting Gtfs by inhibiting their activity and preventing EPS synthesis could impair the virulence of *S. mutans* without threatening their existence or that of other species in the oral cavity. This method preserves the natural oral bacterial flora and does not promote bacterial antibiotic resistance (Zhang et al., 2021).

The most common methods for preventing dental caries include brushing teeth and using dental floss. An ideal strategy is to combine these methods with the regular use of antimicrobial agents, such as chlorhexidine, sodium fluoride, and antimicrobial peptides. However, these chemical agents possess broad-spectrum bactericidal functions, which can disrupt the ecological balance between pathogens and commensal bacteria in the oral cavity. Additionally, the emergence of microbial resistance to these agents is concerning (Philip et al., 2019). The frequent use of chlorhexidine can lead to adverse reactions, including dental calculus and tooth discoloration, making it unsuitable for daily use (Qiu et al., 2020). High-concentration fluorides exert toxic effects, such as fluorosis (Bradshaw et al., 2002), and biodegradation in saliva can reduce the activity of antimicrobial peptides (Mai et al., 2011). Therefore, this necessitates developing non-bactericidal agents that can selectively inhibit cariogenic biofilms while maintaining ecological balance (Nijampatnam et al., 2020). Natural compounds, which are abundant and cost-effective, exhibit lower cytotoxicity, highlighting their feasibility for clinical applications and research focus on dental caries prevention and treatment (Moghadam et al., 2020).

In this study, we established a rapid detection method for Gtf activity to conduct high-throughput screening of a natural product library. We identified tannic acid (TA) as an inhibitor of Gtf activity. Furthermore, we assessed the impact of TA on *S. mutans* growth, biofilm formation, EPS production, and Gtf activity. Our findings provide novel insights into the inhibition of *S. mutans* virulence and methods for the prevention and treatment of dental caries.

## 2 Materials and methods

### 2.1 Strains and chemicals

The experimental strain UA159 of *S. mutans* was obtained from the American Type Culture Collection. This strain was cultivated in brain-heart infusion broth (BHI, Difco, Sparks, MD, United States) at 37°C in an anaerobic incubator (Thermo Fisher Scientific, Inc., Waltham, MA, United States) with 5% CO_2_ to ensure optimal growth conditions. For biofilm formation assays, sucrose (1%, weight/volume) was supplied to the broth. The Natural Product Library was purchased from Selleck Chemicals.

### 2.2 Extracting Gtf

*S. mutans* cultured overnight was diluted at a ratio of 1:10 into 30 mL of BHI media and cultured until reaching the logarithmic growth phase, characterized by an optical density (OD_600nm_) of 0.5 to 0.6. The bacterial cells were removed by centrifugation at 13,000 rpm and 4°C for 10 min. Approximately one-third of the original volume of the supernatant was replaced with anhydrous ethanol (e.g., 10 mL of ethanol for a 30 mL bacterial solution). The mixture was thoroughly mixed and stored at −80°C for 30 min. To prevent tube breakage, the frozen mixture was gently thawed on ice for 10 min. The precipitate was collected by centrifuging the mixture at 4°C and 25,000 rpm for 15 min. Finally, the precipitate was resuspended in 200 μL of phosphate-buffered saline (PBS) to obtain a Gtf suspension (Chen et al., 2021).

### 2.3 Establishing a rapid method for detecting Gtf activity

This method utilizes sucrose as the sole substrate, with T70 glucans serving as the polysaccharide linkage carrier for subsequent production. Gtf catalyzes glucan synthesis from sucrose, and the resulting absorbance value indicates their enzymatic activity. First, 0, 5, 10, 15, 20, 25, and 30 μL of Gtf were dispensed into 96-well plates. Second, 0.2 M sodium phosphate buffer comprising 0.2% T70 glucans (Sigma) and 2.5% sucrose was added to each well. The total substrate volume was 200 μL. Each concentration level was assessed in triplicate. Third, 50 μL of mineral oil was used to seal the sample wells, ensuring system stability. The reaction temperature was maintained at 37°C, and a full-wavelength enzyme-linked microplate reader (Biotek, Epoch, United States) was used to monitor Gtf activity continuously at 540 nm over 24 h. Subsequently, to enhance the screening efficiency of the method, various physicochemical factors influencing the reaction were evaluated, including pH level, reaction time, temperature, and the presence of metal ions. For instance, the absorbance values were monitored hourly using a microplate reader, the type of sugar in the substrate was altered, hydrochloric acid and sodium hydroxide solutions were used to adjust the pH of the reaction solution, different temperatures were set on the microplate reader, and 100 μM of different metal ions (MgSO_4_, CaCl_2_, ZnSO_4_, CoSO_4_, MnSO_4_) were added to the reaction solution. The OD_540nm_ of the reaction was observed under different conditions.

### 2.4 Screening the natural product library

The Natural Product Library—comprising over 1,000 natural molecules—was screened using the established rapid detection method to assess their inhibitory effects on Gtf activity. The stock plates were stored at −80°C, with molecules dissolved in dimethyl sulfoxide (DMSO) (or partially in water) at a concentration of 10 mM (or 2 mM), occupying columns 2 through 11 of each plate. During screening, the plates were thawed and the compounds were allocated to columns 2 through 11 of a 96-well plate, followed by the addition of 30 μg of Gtf extract and sodium phosphate buffer comprising 0.2% T70 glucans and 5% sucrose. The final concentration of each compound was 50 μM. A 0.5% DMSO solution served as the solvent control group, whereas a drug-free solution served as the vehicle control group. A plate reader was used to record the hourly absorbance at 540 nm.

### 2.5 Determining the *Streptococcus mutans* growth curve

For the planktonic bacterial growth assay, the overnight culture of *S. mutans* was cultured in a BHI medium until reaching the logarithmic growth phase (OD_600nm_ = 0.5 to 0.6). The culture was inoculated into BHI broth comprising 50 μM and 100 μM of compound TA at a dilution rate of 1:100. A BHI group without TA served as a control. The cultures were incubated at 37°C for 24 h. The absorbance was recorded every 0.5 h at 600 nm using a microplate reader (Li et al., 2019). This procedure was repeated for the other three compounds: Brazilin, ginkgolic acid, and anacardic acid. Each analysis was conducted in triplicate, and growth curves were plotted.

### 2.6 Determining the MIC50 of *Streptococcus mutans*

The double dilution method was employed. Firstly, *S. mutans* was cultured overnight, diluted 1:10 in fresh BHI liquid medium, and grown to the logarithmic growth phase before being further diluted 1:100 by volume. The original culture was gradually diluted with BHI medium, and *S. mutans* culture was added for incubation. The final concentrations of TA in the media were ensured to be 3,200, 1,600, 800, 400, 200, 100, and 0 μM, respectively. These cultures were dispensed into 96-well plates, including a blank control group containing only pure BHI liquid. The plates were incubated at 37°C in a 5% CO_2_ incubator for 24 h. To quantify the MIC, 100 μL aliquots from the wells were spread in a 10-fold gradient dilution onto BHI agar plates and counted after 48 h of incubation. The MIC was defined as the lowest concentration of the antimicrobial agent that completely inhibited bacterial growth, with the concentration of viable cells being lower than the original concentration. All measurements were repeated six times on different days. The MIC values from all tests were statistically analyzed to determine the concentration of the drug that inhibited the growth of 50% of the strains, which was designated as the MIC50 of *S. mutans* against TA.

### 2.7 Zymogram assays for Gtf activities

The extracted Gtf were incubated in a buffer system comprising 50 μM and 100 μM of TA at 37°C for 3 h. The solution was mixed with loading buffer at a 1:1 ratio and loaded onto two 6% sodium dodecyl sulfate-polyacrylamide gels (SDS-PAGE). One gel was stained with Coomassie Blue R-250 (Sigma) to detect total protein, whereas the other gel was used for zymogram analysis to assess Gtf activity. For zymogram analysis, the gel was immersed in a cold rehydration buffer comprising 2.5% (v/v) Triton X-100 (Sigma). It was incubated at 37°C in 0.2 M sodium phosphate buffer comprising 5% sucrose and 0.2% T70 glucans for 18 h. After incubation, the gel was rinsed twice with cold distilled water to visualize the glucan bands synthesized by Gtf. Images were obtained using a gel image analysis system (Chemidoc MP, Bio-Rad, United States) and analyzed with ImageJ software. Statistical data were normalized to the relative glucan content. Each experiment was repeated thrice to ensure reproducibility and accuracy.

### 2.8 Quantitative real-time PCR

qRT-PCR was employed to quantify the expression of *gtf* genes, with 16S rRNA serving as the internal control. *S. mutans* was cultured overnight, diluted 1:10 in fresh BHI medium, and grown until an OD_600nm_ of 0.5 was reached. Subsequently, *S. mutans* was further diluted 1:100. TA was added to the bacterial suspension to achieve final concentrations of 50 μM and 100 μM, with bacterial suspension without TA serving as the control. The samples were then incubated anaerobically at 37°C for 24 h. Extraction was performed using the MasterPure Complete DNA and RNA Purification Kit (Lucigen) according to the manufacturer’s protocol. A 15 mL volume of the cultured bacteria was centrifuged (4,500 × g, 15 min, 4°C). Subsequently, 300 μL of lysis buffer and 2.5 μL of protease K at a concentration of 20 μg/μl were added to the precipitate. After vortexing for 10 s, the samples were incubated at 65°C for 15 min, with vortexing performed every 5 min. The samples were then placed on ice for 5 min, and 175 μL of MPC protein precipitation reagent was added to the samples, followed by vigorous vortexing for 10 s. The samples were centrifuged (10,000 × g, 10 min, 4°C), and 500 μL of isopropanol was added to the recovered supernatant. The tubes were inverted 30–40 times. After centrifugation (10,000 × g, 10 min, 4°C), the precipitate was collected and washed twice with 70% ethanol. The total nucleic acid was resuspended in RNase-free water. cDNA was synthesized using the PrimeScript RT Kit and gDNA Eraser Kit (Takara) according to the manufacturer’s instructions. Gene-specific primers were designed using the Primer3 online tool. Data analysis was performed using QuantStudio 6 Flex software based on the 2^−ΔΔCT^ method. The experiment was conducted using three independent cultures in triplicate. Each analysis was also performed in triplicate.

### 2.9 Molecular docking analysis

The TA structure file (in Structured Data File format) was retrieved from the PubChem database. It was converted to Protein Data Bank (PDB) format using OpenBabel software and prepared for PDBQT file formatting by adding polar hydrogens and fixing charges in AutoDock Tools 1.5.6. The 3D crystal structure of Gtf was obtained from the RCSB Protein Data Bank and converted to a PDB file. Gtfs were preprocessed in AutoDockTools 1.5.6 by removing water, adding hydrogens, and exporting them as a PDBQT format. After preparing the receptor protein and small molecule ligand, a docking grid box encompassing the entire target protein was generated in AutoDockTools 1.5.6. The parameters were saved as a txt file. Finally, molecular docking analysis was conducted using AutoDock Vina Version 1.1.2, and the molecular docking and its interactions were visualized using PyMOL 2.5.0.

### 2.10 Crystal violet staining

The impact of TA on the formation of *S. mutans* biofilms was assessed using crystal violet staining (Jing et al., 2022). The overnight culture of *S. mutans* was grown in BHI until reaching the logarithmic phase. It was diluted 1:100 in BHIS and added to a 96-well plate. TA was added to each well to achieve final concentrations of 50 μM and 100 μM, with the BHIS group without TA serving as a control. After incubating the plate at 37°C for 24 h, the excess supernatant was removed. The biofilms at the bottom of the wells were washed twice with PBS buffer. These biofilms were fixed with 4% (wt/vol) paraformaldehyde for 15 min. A 0.1% (wt/vol) crystal violet staining solution was added for 30 min. The plate was washed three times with PBS, followed by 33% (v/v) acetic acid solution, which was gently shaken for 30 min. Finally, 200 μL of solution from each well was aspirated and transferred to a new 96-well plate, and the OD_575nm_ was recorded. The crystal violet staining was conducted in triplicate, with each concentration tested in three wells per assay.

### 2.11 Scanning electron microscopy

The surface morphology of *S. mutans* biofilms was observed using scanning electron microscopy, per the previously mentioned culture conditions and experimental setup (Zhang et al., 2020). Circular glass coverslips underwent UV sterilization for 2 h and were placed into a 12-well plate. Each well consisted of diluted *S. mutans* suspension and the respective TA concentration. The plates were incubated at 37°C for 24 h. Subsequently, 2.5% (v/v) glutaraldehyde was added to fix the biofilms. The biofilm-coated cover slips underwent gradual dehydration using different concentration gradients of ethanol solution (30, 40, 50, 60, 70, 80, 90, and 100%), with each gradient lasting 15 min. For observation, a scanning electron microscope (Inspect F, FEI, The Netherlands) was used, with magnifications adjusted to 1,000×, 5,000×, and 20,000×. Three random points were selected on each coverslip for a comprehensive examination.

### 2.12 Confocal laser scanning microscope

Confocal laser scanning microscopy was utilized to observe and quantify bacteria and EPS in *S. mutans* biofilms (Klein et al., 2009). Under the same experimental conditions, the bacterial solution was inoculated into a confocal dish and cultured along with Alexa Fluor 647 dye (Invitrogen) at a final concentration of 1 μM, along with the corresponding compounds. After 24 h of anaerobic incubation in the dark, the excess medium was carefully removed. SYTO 9 stain (Invitrogen) was added for an additional 15-min incubation, maintaining dark conditions. Residual stains were thoroughly washed away with physiological saline to prepare the samples for microscopic analysis. Using a 60× oil immersion objective, the biofilms were observed under a confocal laser scanning microscope (N-SIM, Nikon). For each group, three regions were randomly selected for detailed analysis. Microscopic images were processed using ImageJ software to accurately quantify the bacteria and EPS within the biofilms.

### 2.13 Biocompatibility assays

The biocompatibility of TA was assessed by performing a CCK-8 assay on human gingival epithelial cells (HGE). The HGE cell line was provided by the National Key Laboratory of Oral Disease Prevention and Treatment at Sichuan University. HGE cells were seeded into a 96-well plate at a density of 5,000 cells per well and cultured for 24 h at 37 degrees with 5% CO2. The cells were then incubated with the medium containing 50 μM and 100 μM of TA for 1 h, with the medium without TA serving as the control group. The cells were washed with sterile PBS, and 100 μL of CCK-8 reagent was added to each well. After incubating the plate at 37°C for 3 h, the OD_562nm_ was measured. The experiment was conducted in triplicate and repeated three times.

### 2.14 Statistical analyses

All experiments were conducted in triplicate and repeated thrice. Statistical analysis was conducted using Prism 9.0 (GraphPad Software Inc.) and SPSS 20.0 (SPSS Software Inc.). One-way analysis of variance was used to compare the means among multiple groups, followed by a two-tailed Student’s *t*-test to compare the means between two groups. A *p*-value < 0.05 indicated statistical significance.

## 3 Results

### 3.1 Establishing a rapid detection method for Gtf activity

Because of the extended experimental cycle, complex operational steps involving SDS-PAGE, and numerous factors affecting the outcomes, conventional zymogram assay faces challenges in achieving rapid and high-throughput screening of Gtf inhibitors. Therefore, we developed a rapid detection method for Gtf activity characterized by a shorter experimental cycle, simplified procedures, accurate results, and non-toxic and environment-friendly features.

**Figure 1:**
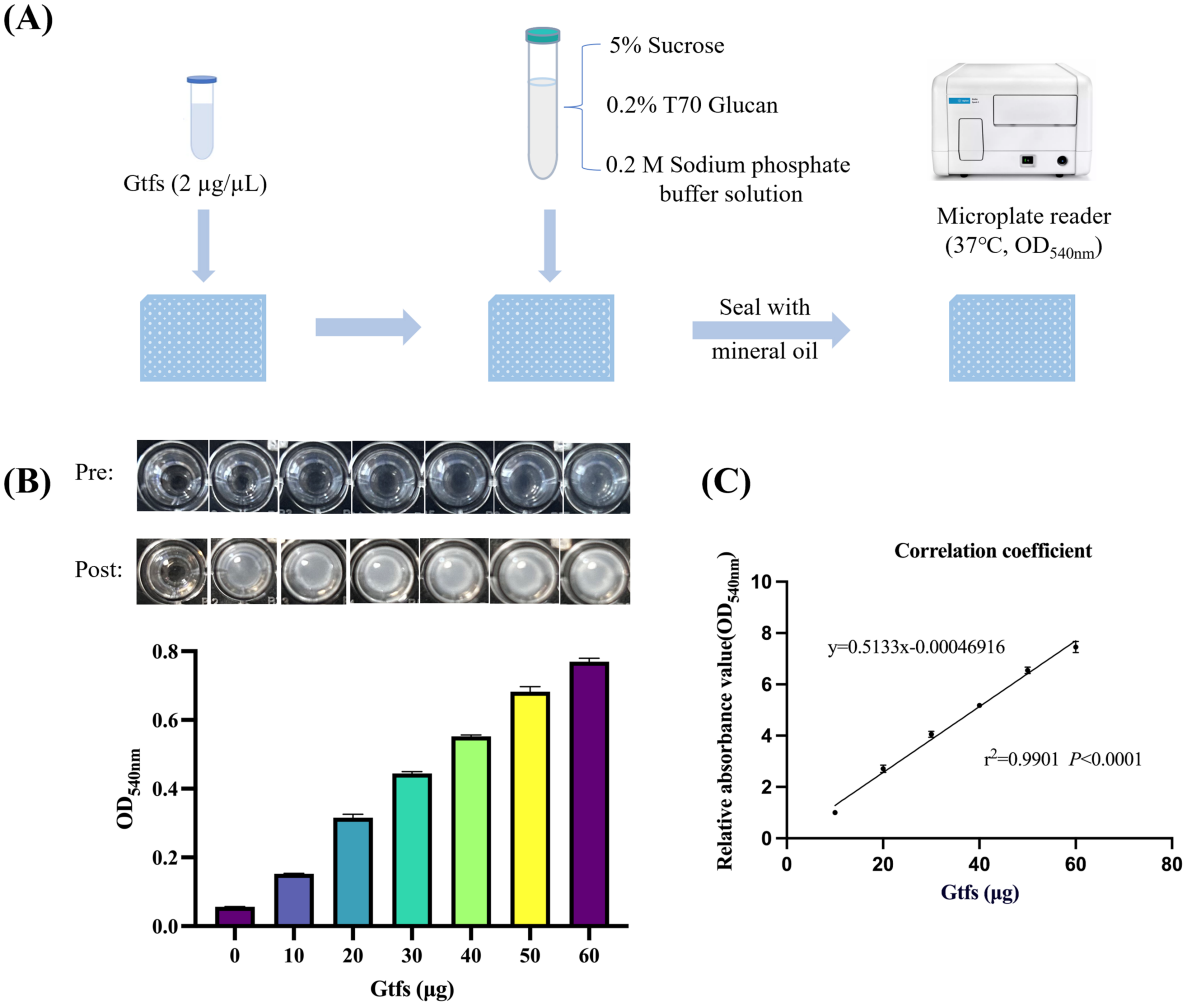
Gtf activity is dose-dependent on its enzyme quantity. **(A)** Flowchart of the rapid detection method designed. **(B)** Well-plate images before and after the reaction (top) and bar charts of enzyme levels and OD_540nm_ (bottom). **(C)** Correlation analysis between the amount of Gtf enzyme and the relative glucan content.

This method uses sucrose as the key substrate and T70 glucans as the subsequent polysaccharide carrier. Gtfs interact with sucrose to catalyze glucan formation, and the resulting absorbance values are used as quantitative indicators of Gtf activity. Figure 1A shows the flowchart for the method design. The amount of glucans generated increased with the Gtf concentration (Figure 1B). A bar chart was plotted based on Gtf concentrations and the measured OD_540nm_. A correlation analysis suggested the dose-dependent correlation between Gtf concentrations and the relative glucan content (Figures 1B,C).

**Figure 2:**
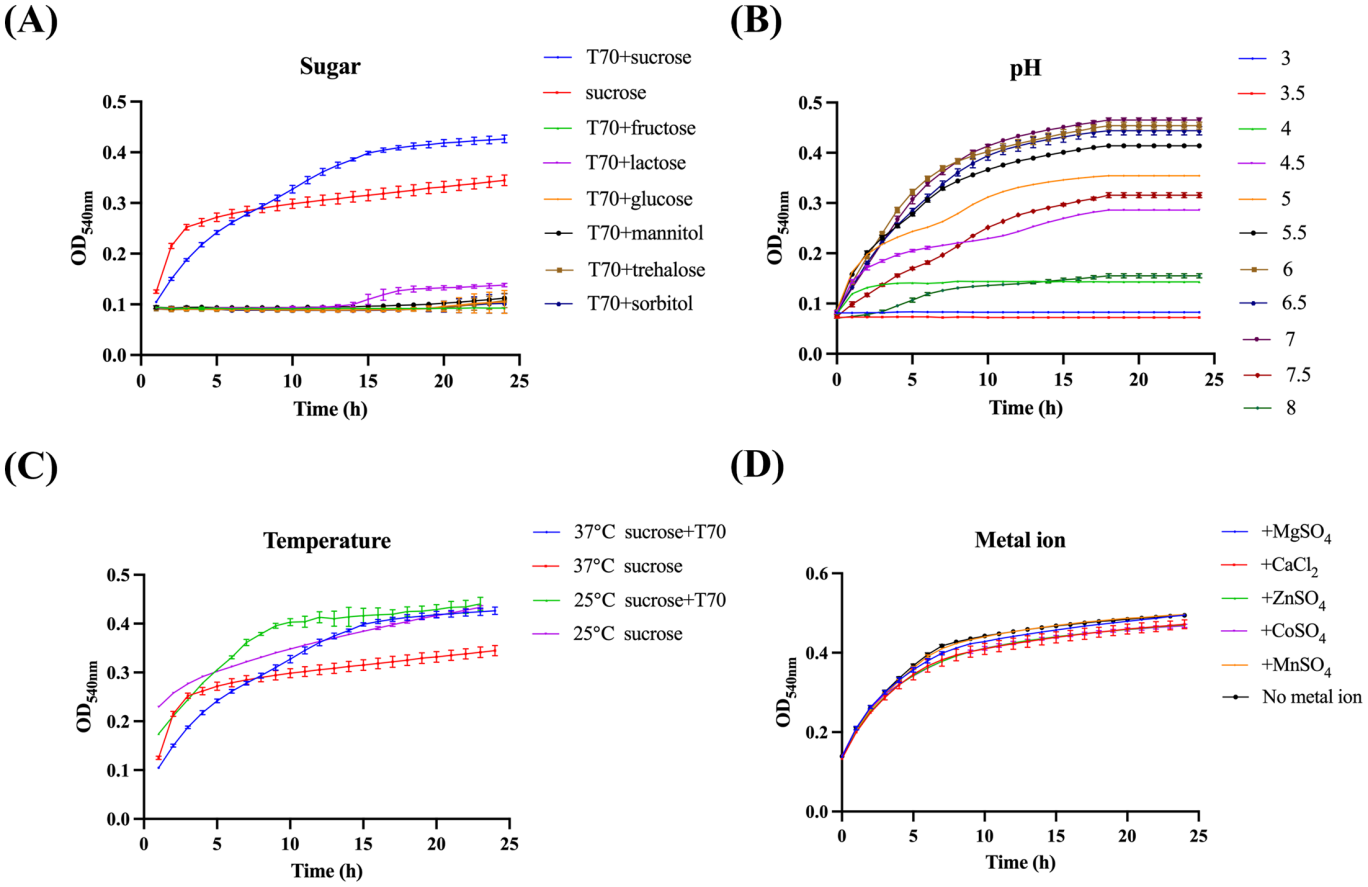
Identifying the ideal reaction conditions for the method. **(A)** Effect of different substrates, **(B)** pH values, **(C)** temperatures, and **(D)** metal ions in the reaction system on Gtf catalytic activity.

To optimize the screening efficacy of our method, we evaluated the impact of physicochemical factors, including pH, reaction time, temperature, and metal ions, on the reaction. At 37°C, Gtf exhibited optimal activity and maximum glucan production upon using T70 glucans and sucrose as substrates (Figures 2A,C). Furthermore, the reaction system achieved peak Gtf activity and glucan production within a pH of 6 to 7 (Figure 2B). Interestingly, the addition of metal ions (100 μM) did not significantly alter Gtf activity (Figure 2D). Based on these observations, we recommend controlling the pH of the reaction system at 6 to 7, maintaining a reaction temperature of 37°C, and allowing a reaction time of 10 to 12 h to yield excellent screening results.

### 3.2 Screening the natural product library to identify compounds that effectively suppress Gtf activity

**Figure 3:**
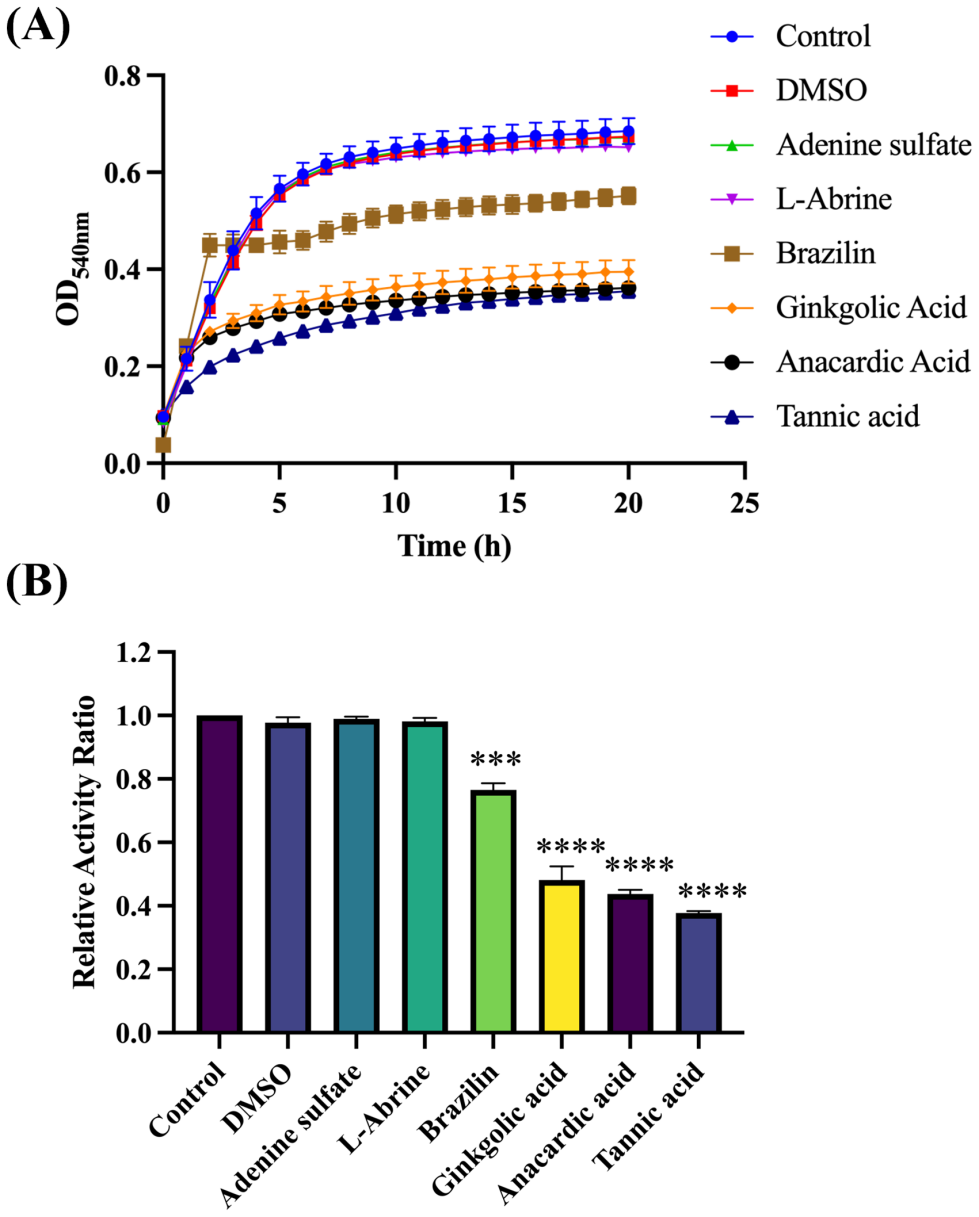
Screening the Selleck Natural Product Library to identify compounds that effectively suppress Gtf activity. **(A)** Plotting growth curves for four effective and two ineffective compounds based on OD_540nm_. **(B)** Relative activity index of four effective and two ineffective compounds, compared with the controls. ***p-value < 0.001, ****p-value < 0.0001.

To identify compounds that can inhibit Gtf activity, we thoroughly screened the Selleck Natural Product Library. OD_540nm_ readings of all wells containing drugs were analyzed and contrasted with those of the blank control wells from identical plates. The difference was quantified as a percentage change relative to the blank control. Of all compounds, the relative activity of four drugs, namely brazilin, ginkgolic acid, anacardic acid, and tannic acid (TA), changed significantly, falling below 70% (Supplementary Figure S1). These compounds necessitate further experimental validation to confirm their inhibitory effects on Gtf activity (Figures 3A,B). Additionally, two ineffective drugs were randomly selected as controls to demonstrate the effectiveness of the method.

### 3.3 Assessing candidate compound cytotoxicity against *Streptococcus mutans*

Under the experimental conditions of drug screening (with a final drug concentration of 50 μM), four candidate drugs were validated by assessing their respective growth curves. TA was the only drug that did not significantly inhibit *S. mutans* growth. Extending the validation to a broader range of final concentrations (100 μM) suggested similar inhibitory effects (Supplementary Figure S2). Only TA appeared to meet our desired experimental outcomes, and, therefore, was selected for further experiments.

### 3.4 TA inhibits Gtf enzyme activity

**Figure 4:**
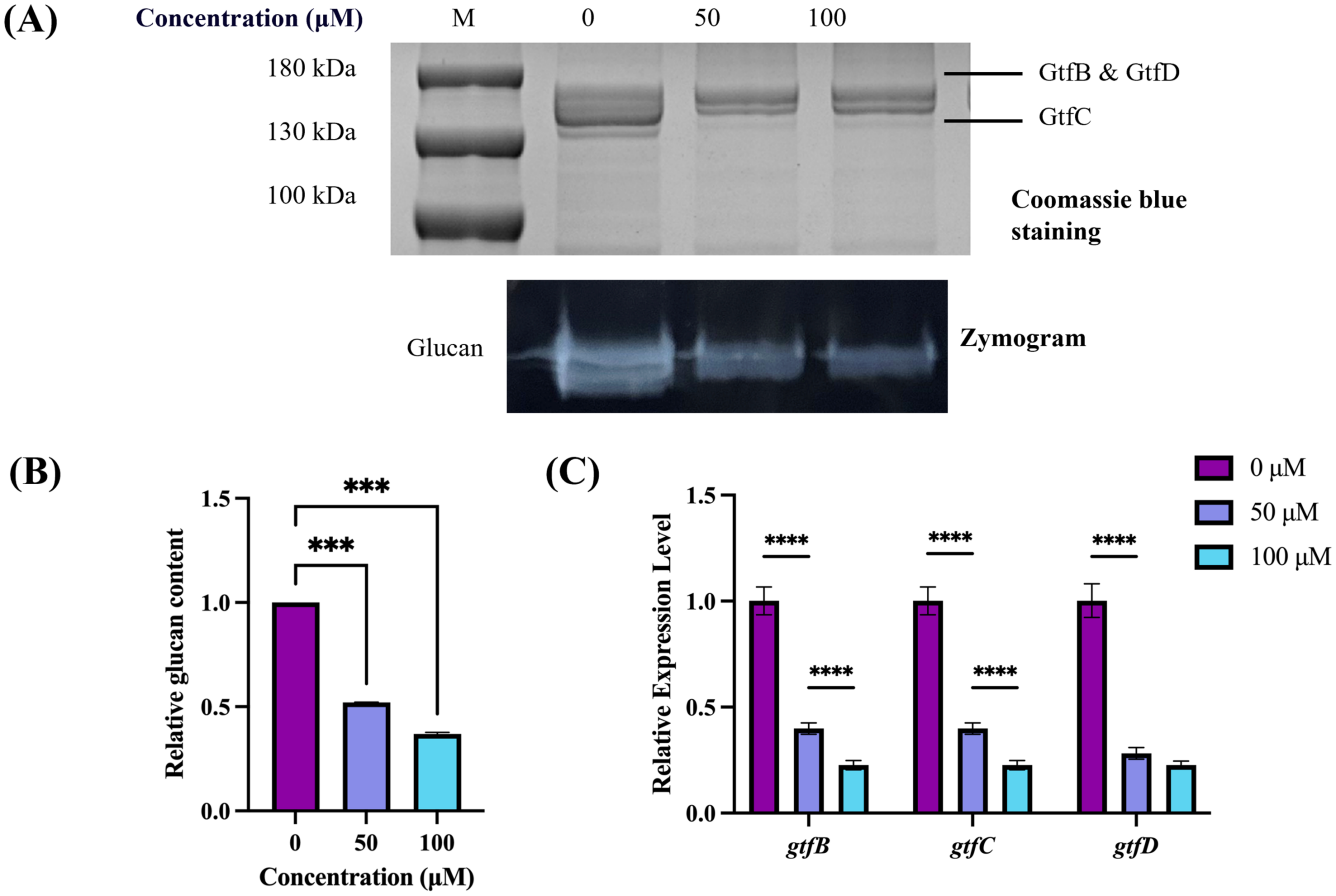
Effect of TA on Gtf activity in *S. mutans*. **(A)** Gtf activity is determined by SDS-PAGE and zymography. The top black band represents the Gtf protein stained with Coomassie Blue, whereas the bottom white band represents the glucans produced by Gtf. **(B)** Band signals are quantified using ImageJ software, and the enzyme zymogram/Coomassie staining values are normalized to the control. **(C)** Differential expression levels of *gtfB*, *gtfC*, and *gtfD* of *S. mutans*. ***p-value < 0.001, ****p-value < 0.0001.

Validation using zymogram assay was conducted to further demonstrate the accuracy of this method and confirm whether TA inhibits Gtf activity. The results exhibited characteristic protein and zymogram patterns of *S. mutans* Gtf (Figure 4A). Specifically, the group without added drugs demonstrated a typical Gtf activity pattern, with two proximal bands at the top representing GtfB (166 kDa) and GtfD (163 kDa) and the lower bands representing GtfC (153 kDa). In the zymogram results (Figure 4A, bottom), white bands depicted the glucans synthesized by Gtf. Their intensity corresponded to glucan production and Gtf enzyme activity. The intensity of glucan bands weakened after TA treatment, suggesting inhibited Gtf activity. Additionally, the quantitative study results were consistent with this observation (Figure 4B). The qRT-PCR study revealed that the expression levels of *gtfB*, *gtfC*, and *gtfD* in the 50 μM and 100 μM TA-treated groups were significantly different from those in the UA159 strain. This indicated that the downregulation of *gtf* genes by TA led to changes in protein expression levels (Figure 4C).

### 3.5 TA interacts with Gtf protein

**Figure 5:**
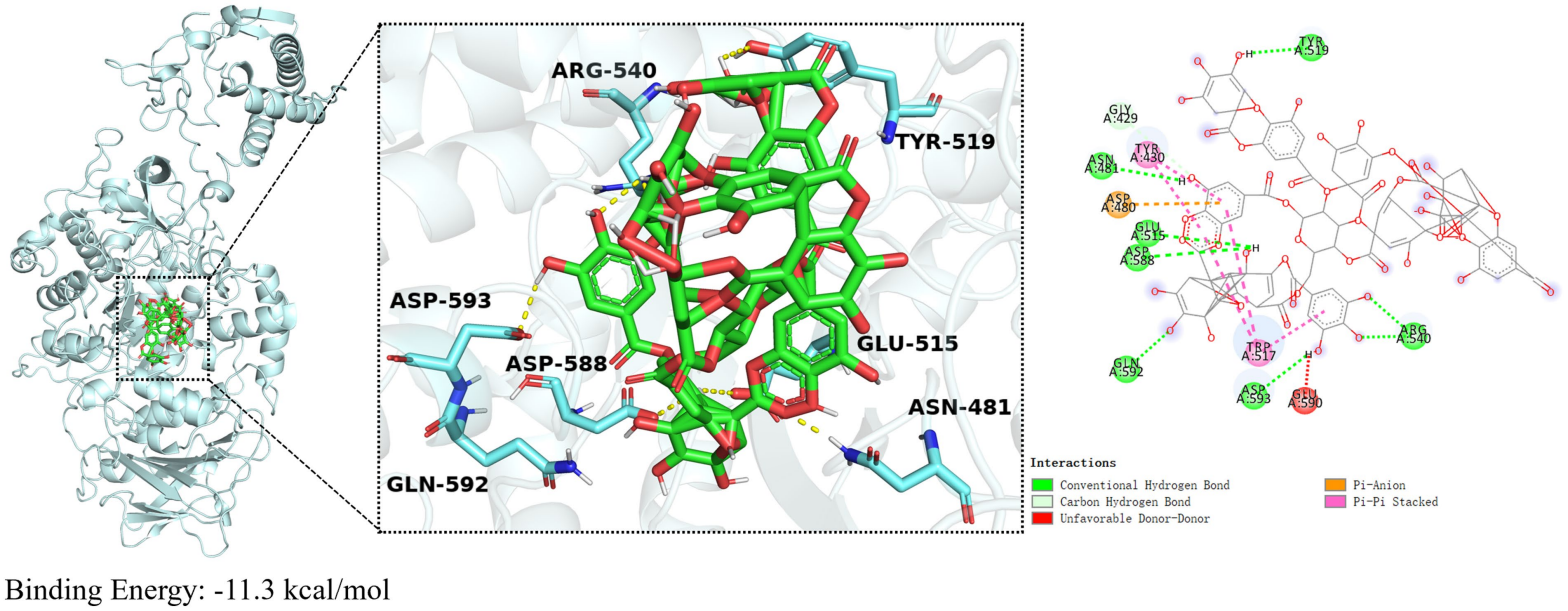
Molecular docking results of TA and Gtf. Molecular docking results are calculated using AutoDockTools 1.5 and visualized using PyMOL 2.5.0.

Molecular docking was utilized to determine the interaction between Gtf and TA, with Autodock Vina facilitating the prediction of binding affinity. Molecular docking experiments, based on the 3D structure, predicted the binding mode and energy (affinity) between the two molecules (Crampon et al., 2022). The docking binding energy between TA and Gtf was −11.3 kcal/mol, indicating a strong binding activity (Figure 5). Figure 5 depicts the 3D and 2D illustrations of molecular docking between TA and Gtf, as well as the related amino acid residues. TA formed hydrogen bonds with TYR519, ASN481, GLU515, ASP588, GLN592, ASP593, and ARG540 on Gtf, van der Waals forces with the GLY429 amino acid residue, Pi-Pi stacked interactions with TYR430 and TRP517, and a Pi-Anion interaction with ASP480.

### 3.6 TA inhibits biofilm formation and EPS production of *Streptococcus mutans*

**Figure 6:**
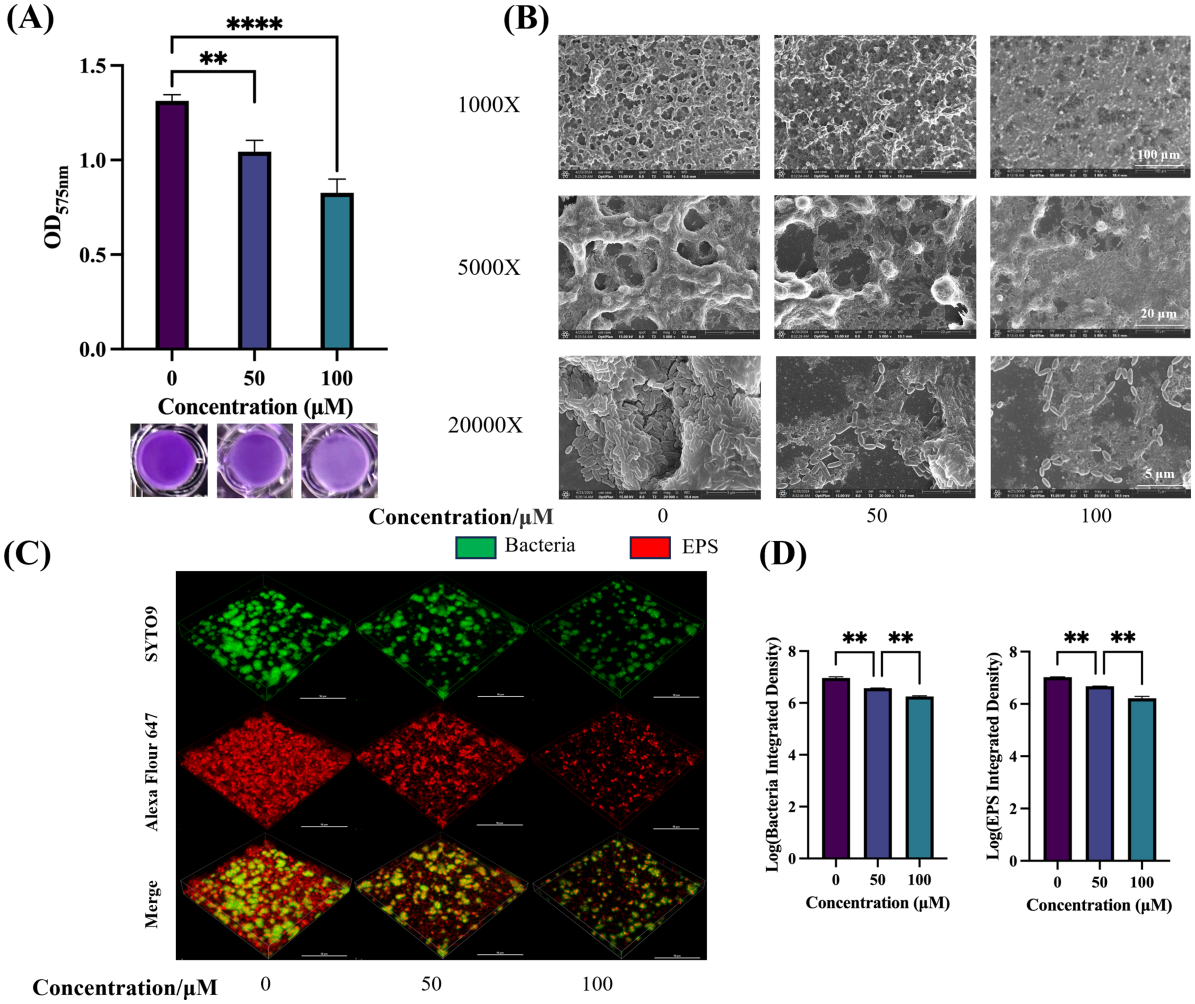
Effect of TA on the biofilm of *S. mutans*. **(A)** Quantification of *S. mutans* biofilm formation at different drug concentrations using crystal violet biofilm assay (OD_575nm_) (top) and biofilm images after crystal violet staining (bottom). **(B)** SEM images of 24-h biofilm of *S. mutans* on glass coverslips, captured at 1,000 ×, 5,000 ×, and 20,000 × magnifications. **(C)** Double-labeling imaging technology is utilized for 3D visualization of the biofilm formed under TA treatment. Specifically, live bacteria are labeled with green fluorescence, whereas EPS is labeled with red fluorescence. This visualization is conducted at 60 × magnification. **(D)** Measuring the quantities of bacterial cells and EPS within the biofilms. Data reflect the mean ± SD derived from three biological replicates. **p-value < 0.01, ****p-value < 0.0001.

The impact of TA on *S. mutans* biofilm formation was assessed using crystal violet staining. Compared with the controls, both 50 μM and 100 μM TA exerted significant inhibitory effects on 24-h biofilms (Figure 6A). SEM was used to display the overall structural changes in the biofilms (Figure 6B). Compared with the controls, biofilms treated with TA displayed reduced EPS with a porous structure.

CLSM was utilized to assess the impact of TA on biofilm structure. Figure 6C presents representative 3D images of 24-h biofilms. Green fluorescence indicates bacteria, while red fluorescence represents EPS. TA treatment resulted in thinner and sparser biofilms, with reduced accumulation of bacteria and EPS on the coverslips. Quantitative data confirmed that TA treatment decreased the biomass of bacteria and water-insoluble EPS within the biofilms, compared with controls (Figure 6D).

### 3.7 TA is biocompatible

**Figure 7:**
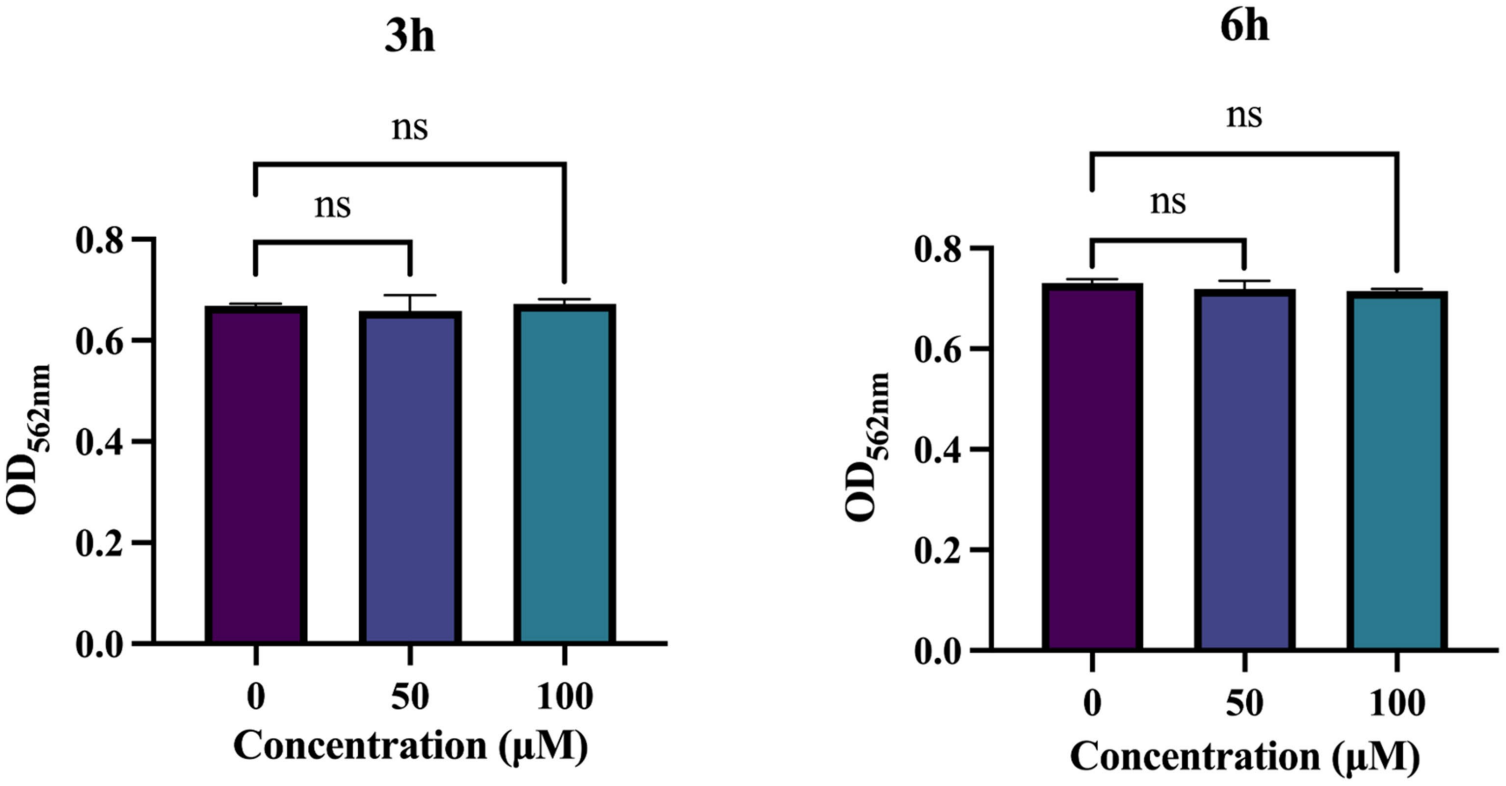
Biocompatibility of tannic acid (TA). Cytotoxicity of TA on human gingival epithelial cells (HGEs) by CCK-8 assay. Results were averaged from 3 separate experiments and are presented as mean ± standard deviation. Statistical significance was determined using one-way ANOVA followed by Dunnett’s multiple comparisons test (ns, not statistically significant compared with the control).

When evaluating the suitability of antimicrobial drugs, their biocompatibility within the body must be taken into account. To investigate the cytotoxicity of TA, we treated HGE cells with a certain concentration of TA for 3 and 6 h to assess its impact on HGE cells. The results showed no significant difference in cell viability between the groups treated with 50 μM and 100 μM TA and the control group, indicating that the concentrations of TA used in this study have minimal toxic effects on human oral cells, suggesting that TA has good biocompatibility (Figure 7).

## 4 Discussion

Dental plaque biofilms promote dental caries (Zhang et al., 2022). Gtf are one of the most important virulence factors of *S. mutans* (Munro et al., 1991). *S. mutans* can synthesize EPS through Gtf; the synthesized EPS promotes its adhesion and accumulation on the tooth surface, increases the stability of biofilms, and enhances its virulence (Bowen, 2002; Ahn et al., 2008). Therefore, targeting the Gtf of *S. mutans* to inhibit biofilm formation is highly effective for the prevention and treatment of dental caries. In this study, we established a rapid detection method for Gtf activity and identified TA, a small-molecule compound, by screening a Natural Product Library. TA inhibits the Gtf activity of *S. mutans* UA159, thereby inhibiting biofilm formation.

Previously, researchers have conducted virtual screenings based on the crystal structure of the glucosyltransferase domain of *S. mutans* GtfC protein to identify Gtf inhibitors (Zhang et al., 2017; Ren et al., 2015). However, compounds selected based on Gtf structure may not inhibit Gtf activity. Zymogram assay, the conventional method for detecting Gtf activity, is relatively complex and has a long experimental cycle, making high-throughput drug screening difficult. Therefore, we developed a rapid detection method for Gtf activity to directly measure the phenotype (OD_540nm_) and enable high-throughput screening of Gtf activity inhibitors.

An additional benefit of Gtf activity inhibitors is their capacity to impede biofilm formation without destroying bacterial cells. The absence of Gtf only reduces adhesion, thereby inhibiting biofilm formation without causing cell death (Koo et al., 2010). In this study, we used the established method to screen and identify four compounds. Three of these compounds inhibited planktonic *S. mutans* growth, whereas TA did not inhibit its growth. Therefore, targeting Gtf activity to impede biofilm formation and maturation may facilitate decreasing the expression of virulence factors and reducing the emergence of bacterial resistance. We validated this finding using zymogram assays; TA inhibited Gtf activity. Additionally, molecular docking studies identified the potential binding sites of TA with the Gtf protein, enabling a deeper understanding of their interaction and offering insights into designing novel TA derivatives.

TA, a natural polyphenolic substance, is primarily derived from plants, such as pomegranates, gallnuts, and chestnut leaves. Its highly complex structure comprises multiple phenolic hydroxyl groups. Because of its broad range of pharmacological effects, such as antioxidant, antitumor, antimicrobial, and anti-inflammatory properties, and interactions with various proteins, TA is widely used in food, industry, and medicine (Chen et al., 2022; Xu et al., 2022). TA can inhibit the growth and biofilm formation of *Agrobacterium tumefaciens*, *Staphylococcus aureus*, and *Pseudomonas aeruginosa* (Jailani et al., 2022; Dong et al., 2018; Siddiqui et al., 2015). Long et al. studied the inhibitory effects of TA on the formation of *Escherichia coli* biofilms and underlying molecular mechanisms, particularly through the influence on the biofilm regulator CsgD (Long et al., 2024). Additionally, a certain concentration of gallnut tannic acid can inhibit *S. mutans* biofilm formation under the influence of shear force (Na et al., 2013). However, the impact of pure TA on *S. mutans* biofilm formation and its specific mechanism of action remains elusive. In our study, TA exhibited anti-biofilm activity against *S. mutans* at concentrations of 50 μM and 100 μM without inhibiting its growth. Within this concentration range, TA demonstrated minimal cytotoxicity toward human oral cells, indicating its biocompatibility. Thus, it holds promise as a novel anti-biofilm agent.

However, the complete interaction between TA and the genetic network responsible for biofilm formation warrants further investigation. Researchers should explore the molecular mechanisms by which TA inhibits biofilm formation. Additionally, oral biofilms are highly dynamic microbial environments, necessitating further studies to explore the impact of TA under real conditions. Zymography only assesses changes in free Gtf activity; variations in cell-associated Gtf activity warrant further research at the molecular level to explore the interaction between TA and cell-associated Gtf.

In summary, we established a rapid method to detect Gtf activity and identified TA as an inhibitor targeting Gtf activity in *S. mutans*. TA can inhibit EPS production and biofilm formation of *S. mutans.* Because of its excellent anti-biofilm properties, this natural compound can be utilized to prevent and treat dental caries.

## Data Availability

The original contributions presented in the study are included in the article/Supplementary material, further inquiries can be directed to the corresponding authors.

## Supplementary material

Figure S1 Screening the Selleck Natural Product Library to identify compounds that effectively suppress Gtf activity.

Figure S2 Validating candidate compounds. Growth curve of *S. mutans* treated with ginkgolic acid, anacardic acid, brazilin, and TA. Compounds at concentrations of 50 μM and 100 μM are incubated with *S. mutans* for 24 h.
