# Supplementary figures and images for: Glucosyltransferase activity-based screening identifies tannic acid as an inhibitor of Streptococcus mutans biofilm
Source: Front Microbiol. 2025 Mar 27;16:1555497. doi: 10.3389/fmicb.2025.1555497 (PMC11984945; doi:10.3389/fmicb.2025.1555497)

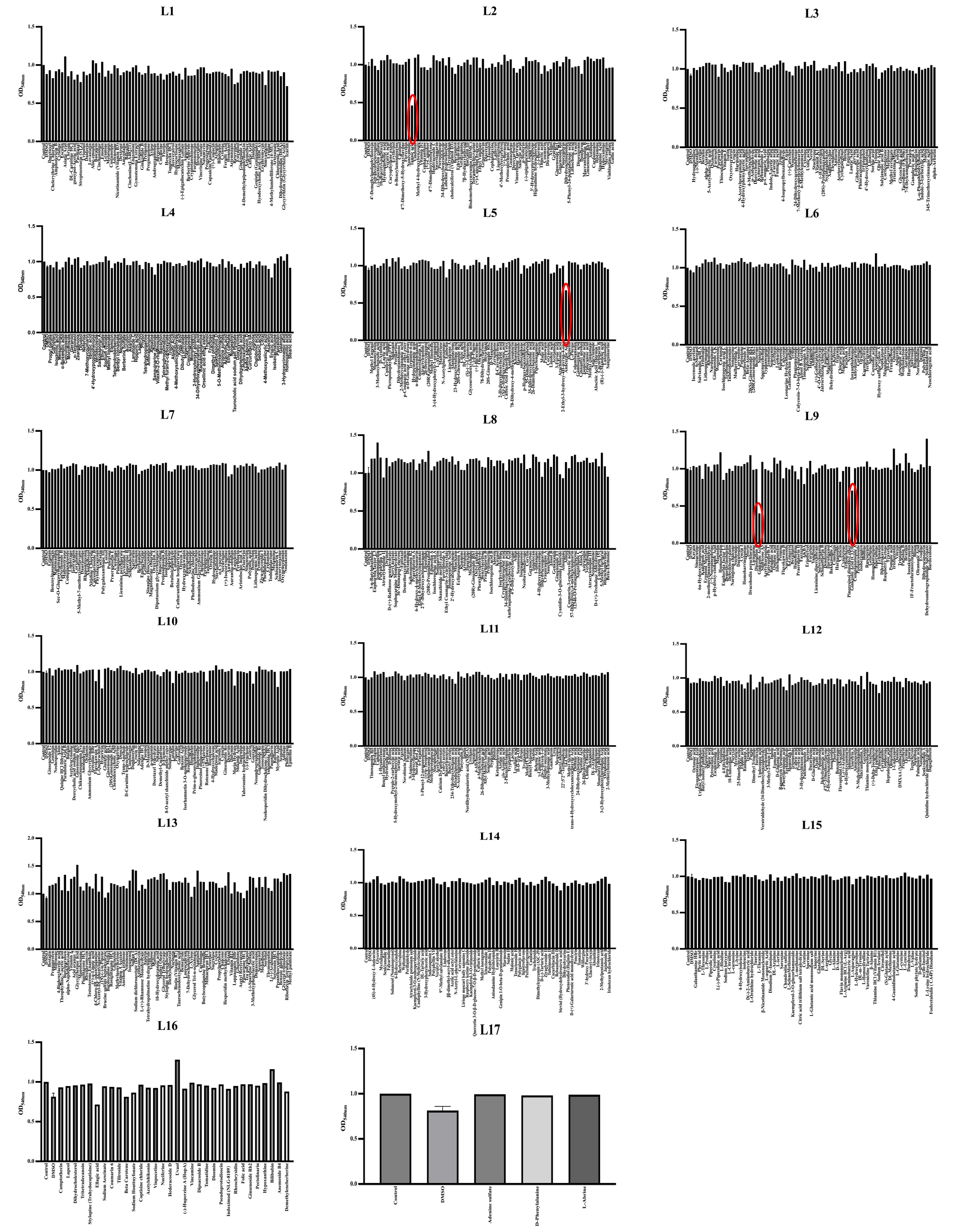

Supplement: Figure S1 — Screening the Selleck Natural Product Library to identify compounds that effectively suppress Gtf activity. [file Image_1.tif]

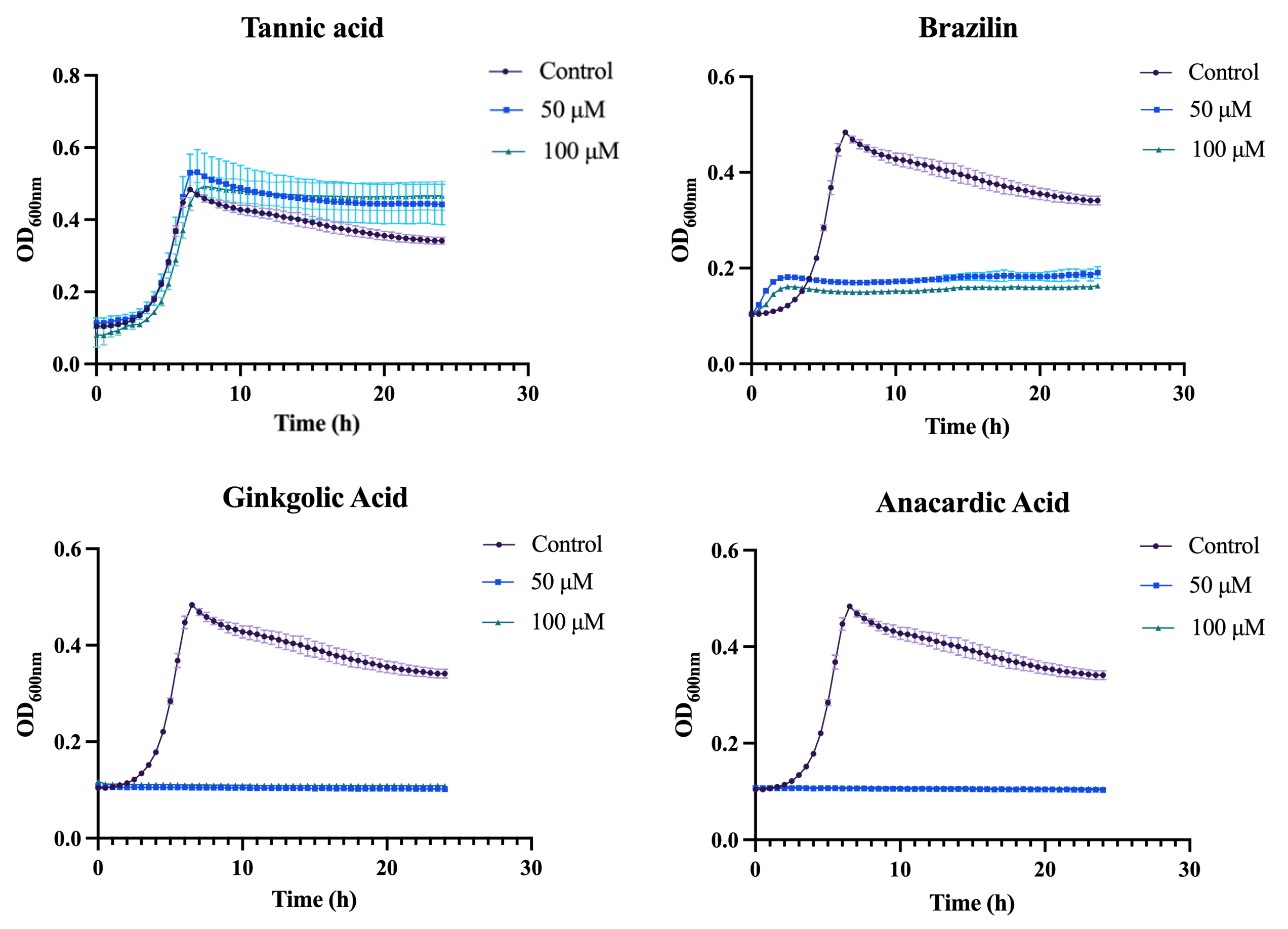

Supplement: Figure S2 — Validating candidate compounds. Growth curve of S. mutans treated with ginkgolic acid, anacardic acid, brazilin, and TA. Compounds at concentrations of 50 μM and 100 μM are incubated with S. mutans for 24 h. [file Image_2.tif]
